# Investigating the effects of a randomized, double-blinded aerobic, resistance, and cognitive training clinical trial on neurocognitive function in older adults with cardiovascular risk factors: the ACTIONcardioRisk protocol

**DOI:** 10.3389/fnagi.2025.1605128

**Published:** 2025-06-23

**Authors:** Louis Bherer, Tudor Vrinceanu, Emma Gabrielle Dupuy, Mathieu Gayda, Thomas Vincent, Pierre-Olivier Magnan, Hanieh Mohammadi, Claudine Gauthier, Christine Gagnon, Simon Duchesne, Kirk I. Erickson, Daniel Gagnon, Frédéric Lesage, Sonia Lupien, Judes Poirier, Marie-Pierre Dubé, Éric Thorin, Martin Juneau, Juliana Breton, Sylvie Belleville, Guylaine Ferland, Flavie Gaudreau-Majeau, Caroll-Ann Blanchette, Paolo Vitali, Anil Nigam

**Affiliations:** ^1^Research Centre and Centre EPIC, Montreal Heart Institute, Montréal, QC, Canada; ^2^Department of Medicine, Université de Montréal, Montréal, QC, Canada; ^3^Research Centre, Institut Universitaire de Gériatrie de Montréal, Montréal, QC, Canada; ^4^Department of Physics, Concordia University, Montreal, QC, Canada; ^5^Department of Radiology and nuclear medicine, Laval University, Québec City, QC, Canada; ^6^AdventHealth Research Institute, Neuroscience, Orlando, FL, United States; ^7^Department of Psychiatry, Université de Montréal, Montréal, QC, Canada; ^8^Department of Psychiatry, McGill University, Montreal, QC, Canada; ^9^Department of Psychology, Université de Montréal, Montréal, QC, Canada; ^10^Department of Nutrition, Université de Montréal, QC, Canada; ^11^Department of Neurology, McGill University, Montreal, QC, Canada

**Keywords:** cognitive prevention, exercise training, multidomain training, cerebrovascular imaging, active control

## Abstract

**Background:**

Lifestyle factors like exercise and cognitive stimulation might help improve cognitive performance in older adults. However, studies investigating this, reported mixed results. Most of the data supporting the benefit of exercise comes from cross-sectional studies, cohort studies, or short intervention studies of 3–6 months with poorly designed control groups. Meta-analyses suggest that longer intervention studies of around 1 year are more likely to show cognitive improvements and changes in brain biomarkers. Moreover, the type and content and optimal dose of the training program that best predict improvement in cognition is still poorly understood. Latest studies suggest that combining cognitive training with exercise training might have an added benefit. Moreover, functional and structural cerebral mechanisms involved are still poorly documented. Finally, few studies have systematically investigated the potential impact that cardiovascular risk factors (CVRF) progression might have on training neurocognitive outcomes.

**Methods:**

159 seniors over the age of 60 with CVRF and no contraindications to exercise will be assigned to one of the three 1-year training programs: (1) Physical exercise intervention (aerobic and resistance exercises); (2) Multidomain intervention (combined cognitive training with aerobic and resistance exercises); or (3) Active control (stretching and toning exercises). All interventions take place 3 times a week, are supervised and individualized to each participant’s profile. Assessments will be administered before, half-way and after the intervention: cognition (primary outcome), cerebral imaging with a focus on cerebrovascular mechanisms (secondary outcomes), and exploratory outcomes (genetic profile, chronic stress biomarkers, metabolic function, inflammation markers, mood, sleep, and diet).

**Discussion:**

The present design uses a 12-month intervention period to maximize the likelihood of identifying the cerebrovascular markers involved in exercise training effects on cognitive performance in individuals with CVRF. Moreover, we measure a series of exploratory outcomes that could also play a role in modulating the effect of the multidomain training on cognition. This will allow an investigation of their potential mediating role on the primary outcomes.

**Clinical trial registration:**

[https://clinicaltrials.gov/] identifier [NCT04962061].

## 1 Introduction

### 1.1 Background and rationale

The proportion of older adults is on the rise in North America, with the number of seniors aged 65 and older growing six times faster than children under 14 years of age (Statistics Canada, 2022). This will lead to a steep increase in the number of individuals at risk for or living with dementia. Worldwide, similar trends imply that dementia prevalence - affecting 47 million people in 2015—is projected to triple by 2050.

One of the main causes behind this rise in dementia prevalence can be attributed to cardio-vascular risk factors (CVRF), such as physical inactivity, diabetes, hypertension, and high cholesterol; their prevalence also increases drastically with age. For instance, 47.2% of Canadians aged 65 and older live with hypertension (Abete et al., 2014). In addition, less than 5% of older adults over the age of 60 accumulate more than 30 min of physical activity per day while more than 90% of them are sedentary for more than 8 h a day (Copeland et al., 2015; Colley et al., 2011). Individuals with CVRF often show impaired cognition, such as attention, executive functions, and memory deficits (Dregan et al., 2013; DeRight et al., 2015), and are at higher risk of developing dementia (Fillit et al., 2008). Moreover, due to the chronic nature of those CVRF their detrimental impact on cognition is more evident in advanced age (Leritz et al., 2011).

Currently, there is no cure for dementia, but evidence suggests that reducing the impact of lifestyle risk factors—in particular, CVRF—can help mitigate the trajectory of cognitive decline. A preventive approach aimed at slowing and halting the development of dementia received strong support in recent years with the publication of major recommendations from leading medical organizations (Livingston et al., 2020; Petersen et al., 2018; National Academies of Sciences, 2017). All reports emphasized the notion that age-related cognitive decline and potentially dementia can be prevented through better lifestyle choices and management of CVRF. They also agreed that further studies are needed to better develop and prescribe lifestyle interventions, as well as to understand the neurovascular mechanisms by which these interventions enhance cognition and how they interact with other factors.

In cognitively healthy older adults, numerous studies suggest that exercise training and cognitive stimulation can help enhance cognitive performance. More precisely, multidomain interventions that include both physical and cognitive training, have shown beneficial effects on cognition in older adults without cognitive impairment and with mild cognitive impairment (Zhu et al., 2016; Lauenroth et al., 2016; Karssemeijer et al., 2017). While the effect of such programs on cognition in individuals with CVRF is not well documented, some meta-analyses suggest that multidomain interventions could lead to larger cognitive improvements in older adults with different health profiles, including those that are healthy, don’t perform physical activity, or that might show metabolic and vascular risk factors (Zhu et al., 2016) but also in patients with cognitive decline (Karssemeijer et al., 2017) relative to exercise training alone. Yet, these meta-analyses highlight the need for conducting studies with multiple arms comparing the efficacy of these combined interventions to individual ones.

The FINGER trial, a large randomized controlled trial investigating the effects of a 2-year multidomain intervention (exercise training, cognitive training and nutritional counseling) observed that cognitive functioning was improved or maintained in individuals with high CVRF (CAIDE score) compared to a control group (no intervention) (Ngandu et al., 2015). However, the design of the FINGER trial does not allow one to determine whether gains are larger in multidomain compared to single-domain interventions. In a more recent study, we reported that an aerobic and strength training exercise program, combined with cognitive training for 6 months, showed larger cognitive benefits compared to either one of the two programs or with placebo conditions for both exercise and/or cognition (Montero-Odasso et al., 2023). This study was unique in comparing all potential combinations of exercise and cognitive training with placebo conditions. The study also suggested that benefits could be observed even in the presence of mild cognitive impairment. While this study observed a positive effect after only 6 months of intervention, scientific reviews suggested that longer interventions of 1-year are more likely to show cognitive benefits (Ludyga et al., 2020). This is particularly true in brain imaging studies which are more likely to show changes in brain structure and function when the intervention is longer (Gomes-Osman et al., 2018).

In fact, several brain imaging studies have suggested that physical exercise induces transient and permanent changes at the structural and functional levels in the brain (Bherer et al., 2013). For example, a seminal study (Erickson et al., 2011) reported increased hippocampus volume bilaterally after a 1-year training program. Another study has showed a link between cardiorespiratory fitness (VO_2_max) and activation in the left dorsolateral prefrontal cortex during a Stroop task (Dupuy et al., 2015) using fNIRS in healthy older women. The study also observed that VO_2_ max was positively associated with blood oxygenation level-dependent cerebrovascular reactivity to CO_2_ in some brain regions (periventricular watershed regions and within the postcentral gyrus), which suggests that preserved vessel elasticity may be one of the key mechanisms by which physical exercise helps to alleviate cognitive aging. In fact, novel studies suggest that the development of dementia and cognitive aging are primarily dependent on specific cerebrovascular health markers both in healthy and individuals with CVRF (Jokinen et al., 2020; Inoue et al., 2023). Specifically, the interplay between cerebral small vessel disease (CSVD) progression and cerebral pulsatility has been proposed as leading mechanisms. While in CSVD pathology regular exercise training is thought to exert an influence, its effectiveness remains a subject of debate in the literature (Shin et al., 2023; Landman et al., 2021). Similarly, the effect of exercise training on cerebral pulsatility is not fully understood due to the limited studies available.

Cognitive training can also induce structural and functional brain changes. For instance, a study (Erickson et al., 2007) showed functional brain changes in frontal regions after dual-task training in healthy individuals. Yet, how these training-induced cerebral changes relate to individuals with CVRF remains to be documented. Brain changes have also been reported with combined or multi-domain interventions. Köbe et al. (2016) performed a 6-month intervention combining aerobic training, cognitive stimulation and an omega-3 supplement in individuals with mild cognitive impairment and observed increased gray matter volume in several brain regions (frontal cortex, angular cortex, precuneus, posterior cingulate cortex) in the training group, while volume decreased in controls. Finally, a recent systematic review (Intzandt et al., 2021) investigating the impact of exercise training and/or cognitive training on cerebral MRI markers, found that different types of exercise training and cognitive training can improve different cerebral MRI markers. This would suggest that a multidomain training intervention could improve cognition by benefiting from the pooled cerebral improvements from all different individual components of the training. However, this remains to be further studied in the same design, using an intervention approach that carefully controls for other confounding factors.

Aging is a heterogeneous process and the health profile of older adults can include multiple medical conditions that can impact the effectiveness of lifestyle interventions. For example, a majority of the older adults over the age of 60 are likely to suffer at some point from cardiac disease, hypertension or arthritis and about a quarter of them are likely to receive a cancer diagnosis (Pilleron and Bastiaannet, 2024; Jafarzadeh and Felson, 2018; Muli et al., 2020; Lettino et al., 2022). Some individuals might suffer only from one condition while other might have multiple ones. Of course, those medical conditions are expected to have a strong impact on the effectiveness of prevention activities. They can also impact the ability to perform certain activities like physical exercise. However, to show that a preventive lifestyle intervention is effective, it is crucial to also know if the intervention has a positive impact before the onset of health deterioration. In other words, it is important to study if regular physical exercise training and cognitive training have a benefit before the appearance of medical conditions and outside of a rehabilitation context. Finally, in order to minimize the bias and accurately study the unique cerebral mechanisms involved in exercise and combined training, a healthy sample will be investigated.

However, even if the older adults don’t have medical conditions, it is important to test if the improvement in cognition could be modulated by various individual factors. For example, it has been reported that exercise intervention effects on cognition and brain functions can be modulated partly by biological sex, genetic risk factors of dementia (APOE-e4 carriers vs. non-carriers) and a polymorphism (Val66Met) within the BDNF gene (Barha et al., 2017). Moreover, age-related change in cortisol, a stress biomarker, is often associated with negative consequences such as increased risks of cardiovascular disease (Whitworth et al., 2005) and cognitive deficits (Lupien et al., 2007). Evidence suggests that physical activity has a positive impact on stress (Childs and de Wit, 2014; Lucertini et al., 2015; Vrinceanu et al., 2019; Molina-Hidalgo et al., 2023). Other subclinical markers like BMI or blood pressure could also modulate the effectiveness of the training and should be explored.

We propose here a study designed to investigate the effect of a physical exercise program, including aerobic and resistance training, alone or combined with cognitive training relative to an active control group on cognitive performance and brain imaging outcomes in individuals with CVRF. The *novelty of this trial* is supported by the following rationale: First, the interventions are 1-year long, which is in line with the literature suggesting that longer exercise trials are more likely to result in cognitive benefits and reveal changes in imagery-related brain biomarkers. Secondly, the two active intervention training programs are designed based on the latest recommendations, and it also includes a cognitive training component. The active interventions are also compared to a well-designed active control exercise group, pursuing high-quality evidence for the effect of physical exercise or combined training. Thirdly, the CVRF profile of our sample will be well monitored with careful medical follow-ups to identify how the health profile interacts with the effect of the intervention. Fourthly, our secondary outcome will include a comprehensive brain imaging toolbox focusing on novel cerebrovascular health markers in addition to the classic imaging markers often studied in conjunction with cognitive aging and exercise training. Finally, the RCT will track multiple individual factors linked to cognitive aging that could be impacted by the interventions to better understand if certain individuals are more or less likely to benefit from exercise or cognitive training.

### 1.2 Objectives

The goal of this trial is to document the impact of a combined physical and cognitive training intervention on neurocognitive functioning in participants with cardiovascular risk factors, while considering individual factors such as sex and specific alleles and mutations in targeted genes thought to modulate the impact of exercise on cognition and brain functions.

The primary hypothesis will test if the multidomain intervention will show higher cognitive benefits (on global cognition, speed of processing, executive functions, and memory) than the physical exercise training or active control after 12 months of treatment. Secondary objectives will assess if the multidomain intervention will show greater brain imaging benefits (on cerebral autoregulation, cerebral vasoreactivity, cerebral pulsatility, and brain structure) relative to the other two groups. Exploratory objectives will assess if the training improvements are modulated by baseline individual difference factors (like genetic profile, biomarkers, vascular health markers, and psychological markers), but also investigate the potential mediating effects of certain variables that can change over the course of the intervention (e.g., vascular health markers, and psychological markers).

### 1.3 Trial design

**FIGURE 1:**
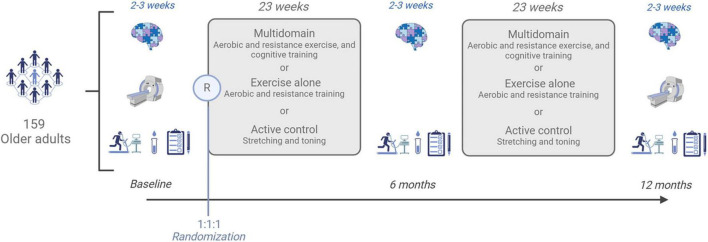
ACTIONcardioRisk study design.

ACTIONcardioRisk is a randomized, double-blind trial of superiority, using an active control group and recruiting physically inactive older adults with cardiovascular risk factors to be allocated to one of three parallel intervention arms: Arm 1—Physical exercise intervention (aerobic and resistance exercises); Arm 2—Multidomain intervention (combined cognitive training with aerobic and resistance exercises); Arm 3—Active control (stretching and toning exercises). The randomization is stratified by sex, with a 1:1:1 training group allocation and it will be done by a staff member uninvolved in this study. The participants’ involvement in the study will be 1 year, with 46 weeks of active training. The participants will be assessed at baseline (T0), 6 months (T1), and 12 months (T2). The testing period will last 2–3 weeks at each timepoint, with three testing sessions per week. During this testing period, no training will be performed. The trial design is illustrated in Figure 1 according to the SPIRIT guidelines (Chan et al., 2013). Please see Table 1 for the World Health Organization trial registration data set.

**TABLE 1:** World Health Organization trial registration data set.

Primary registry and trial identifying number	ClinicalTrials.gov #NCT04962061
Date of registration in primary registry	September 2021
Secondary identifying numbers	n/a
Source(s) of monetary or material support	The Canadian Institutes of Health Research
Primary sponsor	Louis Bherer, PhD, Montreal Heart Institute
Secondary sponsor(s)	Anil Nigam, MD, Montreal Heart Institute
Contact for public queries	louis.bherer@umontreal.ca
Contact for scientific queries	louis.bherer@umontreal.ca
Public title	The impact of combined physical and cognitive training on cognitive performance in seniors with cardiovascular risk factors
Scientific title	Investigating the effects of a randomized, double-blinded aerobic, resistance, and cognitive training clinical trial on neurocognitive function in older adults with cardiovascular risk factors: the ACTIONcardioRisk protocol
Countries of recruitment	Canada
Health condition(s) or problem(s) studied	Seniors with cardiovascular risk factors
Intervention(s)	(1) Combined exercise and cognitive training, (2) exercise training alone, and (3) active control (stretching and toning).
Key inclusion and exclusion criteria	Healthy seniors over the age of 60, with cardiovascular risk factors, without cognitive deficits, and without any counterindication to exercise.
Study type	Randomized, double-blind, active controlled trial of superiority
Date of first enrollment	October 2021
Sample size	159
Recruitment status	Recruiting
Primary outcome(s)	Cognition (global, executive functions, processing speed, episodic memory).
Key secondary outcomes	Neuroimaging (MRI, NIRS, TCD), physical condition, biological markers, cardiovascular healthy, psychological health.
Ethics review	Approved by the Research Ethics Board of the Montreal Heart Institute in 2019.
Completion date	Pending
Summary results	Pending

## 2 Methods

### 2.1 Study setting

All assessments and interventions that are part of ACTIONcardioRisk take place at the Preventive Medicine and Physical Activity Center (ÉPIC Center) of the Montréal Heart Institute. Participants will be recruited from the local older adult community. When participants join the study, they are all registered as members at the EPIC Center, and they are offered an individualized intervention based on the assigned training group.

### 2.2 Eligibility criteria

Potential participants are considered eligible if they are over the age of 60, with normal or corrected vision and normal hearing for their age range. They must be considered physically inactive as measured in accordance with WHO guidelines (WHO, 2023) (i.e., report less than 150-min of moderate-to-vigorous intensity physical activity per week). All participants must also be eligible for an MRI scan (e.g., right-handed, no metallic implants, no claustrophobia). Exclusion criteria involve: dementia or cognitive impairment (i.e., a score of MMSE < 24) (Folstein et al., 1975), or previous clinical diagnosis), uncontrolled or unstable (change in severity or medication) psychological or psychiatric condition within the past 6 months (e.g., depression, bipolar disorder), neurological disease (e.g., Parkinson’s disease, stroke history), non-cardiopulmonary limitation to exercise (e.g., arthritis) or severe exercise intolerance (Physical Activity Readiness Questionnaire—PARQ +), respiratory disease (e.g., severe asthma, COPD, severe COVID-19-related symptoms), cardiovascular disease (including chronic heart failure, symptomatic aortic stenosis, atrial fibrillation, malignant arrhythmias, any documented atherosclerosis disease), automatic implantable defibrillator or permanent pacemaker, and excessive alcohol consumption (report more than 15 drinks/week). All clinical criteria will be first screened by a research assistant using a questionnaire before enrollment and then confirmed with a physician during a medical examination at baseline.

### 2.3 Interventions

All participants follow 46 weeks of intervention, with three training sessions per week of 60–90 min each. Training sessions are performed at the EPIC Center in small groups of up to 10 individuals and are delivered between Monday and Friday by a specialist in kinesiology (for physical training) or neuropsychology (for cognitive training). If desired, the participants have the option to schedule training sessions at home if the training coordinator considers that the participant is sufficiently knowledgeable about the training procedure and safety (see section 2.3.4).

#### 2.3.1 Arm 1—physical exercise intervention (aerobic and resistance exercises)

Participants follow a periodized exercise training program supervised by a certified kinesiologist (with a minimum of a bachelor’s degree). The physical training sessions last 60 min, starting with a 5–10-min warm-up, followed by aerobic and resistance exercises, and ending with a 5–10-min cool-down and stretching period. The aerobic exercises last between 25 and 35 min and are performed mostly on stationary bicycles (other devices used only when participants could not use the stationary bicycle included treadmill or elliptical). Both continuous and high-intensity interval training modalities will be used during the program. The kinesiologist gradually increases the aerobic exercise intensity (in power) throughout the training based on the participant’s feedback. Participants are monitored with a heart rate strap to match the moderate to vigorous intensity zone defined by the American College of Sports Medicine (ACSM) (64–95% of maximum heart rate (HRmax; extracted from the VO2max test), and perceived exertion between 12 and 17 on the 6–20 Borg scale according to training modality). When the exercise intensity is close to the lower bracket on the HRmax or Borg scale, the kinesiologist will propose an increase in power. After aerobic training, 15–20 min of resistance exercises (RE) are performed with a similar individualized progressive increase in intensity. The one maximal repetition (1RM) is assessed for each primary RE movement at baseline, at T6, and at the end of the program. RE intensities during the training are between 40 and 70% of 1RM, for six different types of exercises involving major muscle groups. Each resistance training session is comprised of only three types of exercises of three sets of 6–15 repetitions, with a perceived exertion between 5 and 8 to the 10 pts OMNI scale. The power (aerobic exercise) and load (RE) will be re-adjusted after the T6 evaluation if necessary.

#### 2.3.2 Arm 2—multidomain intervention (combined cognitive training with aerobic and resistance exercises)

The multidomain intervention involves an identical physical exercise program described in the previous section in addition to cognitive training. The cognitive training component takes place before each physical exercise session. Three sessions of 30 min are scheduled each week, including one group session and two individual computerized cognitive sessions. The group session, which takes place once a week, uses strategy-based training adapted from the MEMO + program (Belleville et al., 2018). This group program covers interactive educational lessons on age-related changes in memory and attention and teaches participants different mnemotechnic strategies aimed at improving memory (e.g., face-name association, visual imaging). Some sessions of the group program also aim to help the development of strategies to manage multiple aspects of their daily life, such as anxiety, sleep, and nutrition. The remaining two individual computerized cognitive training sessions focus on the following three components: (a) dual-task training that requires participants to share attention between two concurrent tasks and to maintain and prepare for many alternative responses (i.e., Dual-task) (Lussier et al., 2020); (b) working memory training, which involves the maintenance and constant updating of information in working memory while recalling items presented earlier (i.e., N-Back task) (Boujut et al., 2020); and (c) inhibition and switching training requiring the participants to refrain from giving an automatic response and alternate between following different rules (i.e., Stroop task) (Saillant et al., 2021). During each individual session, participants perform only two of the three different visuomotor tasks (15 min each), each with their respective sets of visual stimuli (e.g., letters, numbers, animals) and matching hand-button symbols. The level of difficulty of each cognitive task is gradually increased over the weeks of intervention to avoid cognitive task automatization and maintain stimulation (Flegal et al., 2019). Participants are instructed to perform as fast as possible while maintaining accuracy. The training also includes online feedback and a histogram of daily performance to encourage improvement. The three tasks will be presented in a fixed order for all participants.

#### 2.3.3 Arm 3—active control (stretching and toning exercises)

Participants in the active control intervention perform three training sessions per week of 60 min each. Each session starts with a 5-min warm-up, followed by a training session that includes muscle toning, balance, and body stretching exercises (light intensity). The proposed exercises were selected from a panel of 30 exercises including balance and muscle-toning exercises, yoga and Pilates sequences. To diversify, muscle-toning exercises might be performed with dumbbells and resistance bands. The intensity of stretching and toning sessions is monitored with a heart rate chest strap and should not exceed the light intensity zone defined by the ACSM (64% of HRmax or less, and a Perceived Exertion lower than 11 on the 6–20 Borg scale).

#### 2.3.4 Home-based training sessions

To remotely conduct their training on the dedicated app and website, participants who want to perform home-based training sessions are required to possess a smartphone, a computer, or a tablet connected to the internet. At the beginning of each month, training sessions at home are scheduled with the kinesiologist and the cognitive training coordinator (for the combined group). Home-based training sessions respect the guidelines of the ACTIONcardioRisk training programs highlighted above. During the first in-person training sessions at the EPIC Center, participants are trained to read heart rate monitors and recognize their perceived effort using the Borg scale. Participants are also trained to master RE and stretching movements and postures and to perform them properly without supervision. A heart rate monitor belt is lent to the participants for the intervention duration to collect continuous heart rate, energy expenditure, and the duration of physical exercise sessions. If the participants are considered sufficiently autonomous by their kinesiologist, they will be allowed to perform all training sessions from home. The compliance of the home-based cognitive training and physical exercise with ACTIONcardioRisk guidelines will be monitored on a weekly basis by the training coordinators on the dedicated app and website. In the multidomain arm, the participants performing strategy-based cognitive training sessions from home will be able to join the group session using a videoconference program. A weekly verification of the execution of the computerized cognitive training sessions is carried out by a research assistant, and a phone follow-up is conducted with the participant as needed. The kinesiologist will check the recorded physical activity training data regularly and a telephone contact is made if they do not adhere to the training program or if changes to the training program need to be implemented.

### 2.4 Outcomes

Information concerning demographics, chronic diseases, comorbidities, medications, medical history as well as anthropometric measurements (e.g., height, weight, waist circumference) are collected during a medical examination at baseline. The study physician will also monitor clinical outcomes at T0 and T2. At each timepoint, the neurocognitive functioning of participants will be assessed using cognitive tests (neuropsychological tests and computerized cognitive tasks) and the cerebrovascular functioning and structural brain imaging will be assessed through neuroimaging approaches (MRI, functional Near-Infrared Spectroscopy – fNIRS, and transcranial Doppler – TCD) before and after the intervention. A comprehensive assessment of physical functioning and body composition (cardiorespiratory fitness, mobility, balance, muscular strength, bioelectrical impedance analysis), cardiovascular and metabolic functions (arterial stiffness and endothelial function, blood draws), biological markers, and self-reported mood, anxiety, and health-related quality of life, will also be performed. All assessments are synthesized in Table 2.

**TABLE 2:** Schedule of assessments.

	Preliminary call	First visit	12 months																		
			Pre-training assessments (T0)							Mid-training assessments (T6)					Post-training assessments (T12)						
**Session**	A	B	1	2	3	4	5	6	7	1	2	3	4	5	1	2	3	4	5	6	7
**Duration (min)**	45	90	125	110	90	110	125	60	150	95	110	120	105	35	95	110	120	105	125	60	150
**Procedure**																					
Consent form	x																				
PARQ +	x																				
Blood draw				x							x					x					
Cortisol		*^2^								*^2^					*^2^						
Gut microbiome		x													*^3^						
Accelerometry		x																			
Medical evaluation			x																		
Hearing assessment			x																		
Questionnaires			*^1^							*^1^					*^1^						
**Cognition**																					
MMSE		x																			
MOCA		x									x					x					
Paper-pencil Assessments				x							x					x					
Computerized tasks 1			x							x					x						
Computerized tasks 2						x							x					x			
Computerized tasks 3							x							x					x		
**Functional status**																					
Mobility		x										x					x				
Functional		x										x					x				
Anthropometric measures			x							x					x						
VO2 max			x							x					x						
**Cardiovascular health**																					
Flow-mediated dilation									x												x
Central artery stiffness									x												x
Vascular reactivity									x												x
**Neuroimaging**																					
MRI							x	x											x	x	
Spectroscopy					x							x					x				
Transcranial Doppler (TCD)						x							x					x			

*_1_Questionnaires will always be given on the first session of every testing period.

*_2_Cortisol material will be given at the first visit and on the first session of T6 and T12 testing periods.

*_3_Gut microbiome material will be given on the first session of T12 testing.

#### 2.4.1 Primary outcome—cognition

Cognitive functioning is assessed using a battery of validated paper-pencil and interview-based neuropsychological assessments and computerized cognitive tasks. All assessments are performed under the supervision of a neuropsychologist or a trained psychology student. The neuropsychological tests are administered in a fixed order: MoCA (Nasreddine et al., 2005), Rey auditory verbal learning test—RAVLT (Lezak et al., 2012), Digit Symbol Substitution Test—DSST (Jaeger, 2018), Stroop Color-Word Interference Test—SCWIT (Jensen and Rohwer, 1999), Phonological and Semantic Fluency from the D-KEFS battery (Letters P, T, L – 60 s maximum) (Delis et al., 2001), Trail Making Test—TMT A and B (Tombaugh, 2004), Digit Span and Similarities subtest from the WAIS-IV (Wechsler, 2008). All the above tests are validated and normalized for an older adult population (Lezak et al., 2012; Strauss et al., 2006).

First, the Montreal Cognitive Assessment (MoCA) score will measure global cognition (Nasreddine et al., 2005). Then, the executive functions, processing speed, and episodic memory will be assessed by three composite Z-scores calculated from specific components of the neuropsychological tests, as it has already been done in the past (Desjardins-Crépeau et al., 2014). The composite Z-scores will be computed by averaging the Z-scores of tests and sub-tests assessing the same cognitive functions. The processing speed score includes: DSST score, time to complete SCWIT naming and reading conditions, and time to complete TMT part A. The executive score includes time to complete inhibition and switching conditions of the SCWIT, and time to complete part B of the TMT. The memory score includes the following components from the RAVLT: total number of words recalled in 5 trials, immediate recall after interference, delayed recall, recognition, and false recognitions. Their internal consistency will then be verified using Cronbach’s alpha (Ferketich, 1990).

Computerized cognitive tasks are also completed, including a dual-task (Lussier et al., 2020), a Stroop task (Saillant et al., 2021), and an N-back task (Boujut et al., 2020). The response time to the computerized tasks is recorded in milliseconds. Subcomponents of each computerized task will allow for the dissociation of attentional control mechanisms from processing speed. To minimize practice effects, a trained and a transfer version (i.e., using different stimuli) of each computerized task is presented in a counterbalanced order between the participants via a Latin square procedure. Previous studies have validated this approach and showed that cognitive-training improvements can be detected through the transfer task (Vrinceanu et al., 2022). The participant’s equipment (i.e., computer or tablet) for the computerized tasks is documented, and participants must use the same equipment across all testing sessions.

#### 2.4.2 Secondary outcome—neuroimaging

The neuroimaging data will be extracted from an MRI scan, a combined NIRS/TCD recording, and an fNIRS recording. The secondary variables of interest are (1) cerebral autoregulation of the frontal cortical region and of the middle cerebral artery (NIRS/TCD + plethysmography), (2) cerebral vasoreactivity of the whole brain (MRI), (3) cerebral vasoreactivity of the prefrontal cortex and of the middle cerebral arteries (NIRS/TCD), (4) cerebral pulsatility of the prefrontal cortex and the middle cerebral arteries (TCD), (5) cerebral activity (fNIRS), (6) brain morphology (structural MRI), and (7) changes in brain small vessel disease (pathological MRI).

##### 2.4.2.1 Magnetic resonance imaging

The MRI recording (Table 3) is made over two separate visits using a Siemens 3T Skyra MRI scanner equipped with a 32-channel array coil. The recording combines anatomical MRI and arterial spin labeling (ASL) MRI to assess cerebrovascular reactivity. In addition, structural markers of cerebral microangiopathy standardly used in the literature are also recorded (e.g., lacunes, microbleeds, white matter hyperintensities). To do so, multi-contrast MRI (T1w, T2w, FLAIR, susceptibility) is acquired in compliance with the STandards for ReportIng Vascular changes on nEuroimaging (STRIVE) (Wardlaw et al., 2013).

**TABLE 3:** MRI acquisition sequences.

Sequence	Resolution	Scan duration	Purpose
T1 MP-RAGE	1 × 1 × 1 mm	7 min	Morphometry, Gray/White matter segmentation
T2 TSE	1 × 1 × 1 mm	4 min	lacunes, perivascular spaces, old infarcts
T2 SWI	1 × 1 × 3 mm	4 min	Microbleeds
DTI	2 × 2 × 2 mm, 64 dirs	14 min	Recent white matter lesions, fractional anisotropy, connectome
ASL Hypercapnia	4 × 4 × 4 mm. TR = 4 s	14 min	Vasoreactivity: variations of CBF induced by hypercapnia

For the breathing manipulation during MRI ASL hypercapnia, the participant will be asked to breathe three pre-mixed gases: a hypercapnic mixture (5% CO_2_, 21%O_2_, and 74% N_2_), a hyperoxic mixture (75%O_2_ and 25% N_2_) and carbogen (5% CO_2_ and 95%O_2_). Participants will be asked to breathe each gas for 2 min, preceded and followed by 2 min of air breathing. The participant will wear a re-breathing face mask connected to an automated gas delivery system. The mask has a sealing contour to prevent leakage and mixture with outside air. The flow rate will be set at 20 L/min. End-tidal CO_2_ and O_2_ concentrations will be measured using the Thornhill Respiract gas monitoring equipment. The participant will be asked to try to mask outside the MRI machine before the beginning of acquisitions to ensure the comfort of the breathing manipulation. Comfort will be assessed using the scale dyspnea scale by Banzett et al. (2015). Participants reporting a discomfort rating of 5 or above will be excluded from this sequence.

##### 2.4.2.2 Near infrared spectroscopy/transcranial Doppler combined recording

A combined NIRS/TCD assessment is used to record cerebral autoregulation and pulsatility. The cerebral autoregulation assessment involves the participant resting in a supine position for 5 min, then standing up for 5 min, while undergoing simultaneous recordings of TCD, NIRS, ECG and continuous arterial blood pressure (finger plethysmograph, Finapres NOVA). For the NIRS/TCD cerebrovascular reactivity measure, the participant is asked to breathe a controlled gas mixture through a mouthpiece, alternating between room air and a hypercapnic mixture (5% CO_2_, 21% O_2_, and 74% N_2_). The session lasts 12 min with alternating 2-min blocks of normal and hypercapnic conditions. While the TCD signal is recorded, the end-tidal O_2_ and CO_2_ concentrations are tracked by a gas analyzer (Gemini O_2_/CO_2_ monitor). Hypercapnic blocks are manually administered by opening/closing a pre-mixed gas cylinder. The values obtained will be related to CVR measures obtained by MRI as a multimodal bridge. The NIRS/TCD system is also employed to assess the regulation of CBF with respect to variations of blood pressure (commonly referred to as static cerebral autoregulation (Donnelly et al., 2015), and the pulsatility that reflects arterial elasticity (Tan et al., 2017). During the NIRS/TCD procedure, a concurrent electro-cardiogram (GE TRAM 3-leads ECG or Finapres NOVA 3-leads ECG add-on) is also recorded to identify cardiac cycles. These cycles are then used to extract diastole and systole amplitudes and compute pulsatility indexes from both NIRS and TCD traces.

##### 2.4.2.3 Functional near infrared spectroscopy

Participants complete an executive function task (Stroop) while being monitored by fNIRS at all-time points, using the Brite23 system from Artinis. The experimental setup consists of 23 sensors (optodes) placed over the prefrontal cortex and driven by a wireless device. Note that the Stroop task differs from the one used for neuropsychological testing as it is optimized for neuroimaging. During this version of the fNIRS Stroop task (10 min), the participants will be asked to identify either the color or the meaning of words as fast as they can. The trials will be categorized as naming (the word is colored, and its spelling is not a color), inhibition (the word spells a color name and is displayed with a color not matching its meaning) and switching (alternation between color identification and word reading). Trials are grouped into blocks of 12 trials, with a presentation rate of 2.5 s. Before each block, an instruction is displayed to indicate the trial type. Each block is followed by 20 s of rest to allow a return to baseline. This testing session is comprised of 4 blocks of each type, for a total of 12 blocks. Neurovascular coupling is assessed during this session.

#### 2.4.3 Tertiary outcomes

##### 2.4.3.1 Physical condition

The cardiorespiratory fitness, body composition, balance, lower and upper limb muscular strength, as well as mobility of the participant, is assessed using standardized and validated methods. Cardiorespiratory fitness is assessed using a VO_2_ Peak test (Gayda et al., 2017) on an electro-mechanically braked bicycle ergometer (Ergoline 800S, Bitz, Germany). Subjects have to maintain a constant pedaling cadence between 60 and 80 revolutions per minute. The test begins after a 3-min warm-up phase at 20 W. Depending on age, sex, and cardiovascular status of the patient, initial workload is individualized and increased by 5–15 W every min until exhaustion. Strong verbal encouragement is given throughout the test. Gas analyzers will be calibrated before each test using a gas mixture of known concentration (15% O_2_ and 5% CO_2_) and ambient air. Participants breathe through a facemask connected to a turbine with low resistance. The turbine is calibrated before each test using a 3-L syringe at several flow rates. Electrocardiographic activity is monitored continuously using an ECG (Marquette, Missouri) and blood pressure is measured every 2 min using a sphygmomanometer. Cardiopulmonary recordings are made every four respiratory cycles during testing and then averaged over 15 s. The maximal exercise test is continued until one of the following criteria are reached: (A) A plateau of VO_2_ despite an increase of power, (B) an R.E.R > 1.1, (C) measured maximal heart rate attaining 95% of age-predicted maximal heart rate, (D) inability to maintain the cycling cadence, (E) subject exhaustion with cessation caused by fatigue and/or other clinical symptoms (dyspnea, abnormal BP responses, or ECG abnormalities that will require exercise cessation). The highest VO_2_ value reached during the exercise phase is considered as the VO_2_Peak, and peak power output is defined as the power output reached at the last fully completed stage.

A bioimpedance analysis using a Tanita system (model 418 C, Japan) is also performed to assess the *body composition* (e.g., total and trunk fat mass, total fat-free mass). *Balance* and *lower limb muscle* strength are assessed by a one-leg balance test and a Five-Time-Sit-to-Stand test, respectively. The *grip strength* is measured for both arms with the help of a hydraulic hand dynamometer. Finally, mobility is assessed using the 10-meter walk test and the Timed Up-and-Go performed at spontaneous and fast walking speeds. These mobility and functional tests are commonly used in a clinical setting and recommended by the Canadian Consortium on Neurodegeneration in Aging (Montero-Odasso et al., 2019). Finally, the amount of physical activity is also recorded using an accelerometry tracker (e.g., Actigraph) worn for 7 days at baseline, before the start of any study visit.

##### 2.4.3.2 Cardiovascular health

General medical data is collected at the first visit, and detailed medical data (including medical history) is collected during the dedicated appointment with a study physician. The number of cardiovascular risk factors is assessed based on the presence of hypertension, dyslipidemia, diabetes, obesity (i.e., waist circumference), active smoking (i.e., one cigarette or more per day), physical inactivity, and family history of premature CVD (i.e., before 45 years for men and 55 for women).

Peripheral endothelial function of the brachial artery: Flow-mediated dilation is quantified by measuring brachial artery blood velocity and diameter by ultrasound (uSmart3300, Terason) with a linear array transducer (5–12 MHz) prior to and following 5 min of forearm ischemia. All procedures are performed according to recent guidelines (Zeki et al., 2014).

Central artery stiffness: Carotid femoral pulse-wave velocity is measured according to recent American Heart Association guidelines (Townsend et al., 2015). Arterial blood pressure tracings are recorded continuously and simultaneously from the carotid and femoral arteries using a pencil tip tonometer. Using the foot of the pressure waveforms, transit time is determined by the delay in arrival of the pulse wave at the carotid and femoral measurement sites. The travel distance of the pulse wave is estimated from distances taken with a measuring tape on the body surface.

##### 2.4.3.3 Biological markers

In an exploratory manner, this trial will collect biological markers of stress, metabolic function, as well as neurotrophic and genetic markers, using diurnal salivary cortisol, blood samples and the gut microbiome. Due to the potentially uncomfortable procedure of collecting the gut microbiome this assessment is made optional.

The salivary diurnal *cortisol* secretion is sampled on three non-consecutive days, at the time of awakening, 30 min after, 60 min after, at 2:00 pm, 4:00 pm, and 9:00 pm. The participants use Salivettes (Sarstedt, Ville St-Laurent) to collect their saliva, and any relevant information related to their collection is reported in a logbook (e.g., time of awakening and bedtime, time of collection). The data collection, manipulation, and interpretation will be done according to the guidelines (Stalder et al., 2016).

A fasting *blood sample* is also collected from all participants to measure lipids profile, glucose, HbA1C, insulin, creatine, electrolytes, high-sensitive c-reactive protein, ApoB, plasma human BDNF concentration (by enzyme-linked immunosorbent assay). DNA and RNA extraction and sequencing are also planned (for future transcriptome investigations, and to generate high quality genome-wide genotyping data that include the APOE and BDNF genes). The blood draw is done fasting by a nurse in the morning.

Gut *microbiome* is assessed using fecal samples collected at baseline and at the end of the intervention (Koblinsky et al., 2023). The participants do the collection at home, after being instructed by a research assistant. The preparation and DNA extraction is done immediately after receiving the samples, then they will be stored at −80°C until further processing.

##### 2.4.3.4 Questionnaires

Validated self-reported questionnaires assess mood, anxiety, health profile, and health-related quality of life of participants. Participants fill the Perceived Stress Scale (Cohen et al., 1983), the Geriatric Depression Scale (Yesavage et al., 1982), the State-Trait Anxiety Inventory (Spielberger et al., 1970), the 12-item Short Form health-related quality of life survey (Ware et al., 1996), the Pittsburgh Sleep Quality Index (Buysse et al., 1989), the Short Diet Questionnaire (Gilsing et al., 2018), the Social and Community Involvement Questionnaire (Richard et al., 2009), the Physical Activity Scale for the Elderly (baseline only) (Washburn et al., 1993), the Short Form Bem Sex-Role Inventory (baseline only) (Bem, 1974), the long COVID symptom and impact questionnaire adapted from Tran et al. (2022) and WHO’s Global COVID-19 Clinical Platform (WHO, 2021). Cognitive reserve is also measured using years of education and a modified version of the Rami and colleagues’ cognitive reserve questionnaire (baseline only) (Rami et al., 2011) adapted for French and English by the CIMA-Q team (Belleville et al., 2019).

### 2.5 Participant timeline

First, a research team member performs the initial contact over the phone. During this call, the study structure is introduced, and a pre-screening of potential participants with no major physical or medical limitations to exercise is done. Interested individuals who meet these two main criteria are then assessed for eligibility. Immediately after the pre-screening call, the interested and eligible participants will receive the information and consent form (ICF). During a preliminary call, the ICF will be further explained in detail, and oral consent will be obtained. Following this, the participants will be screened for major physical limitations to exercises (PARQ +). After obtaining written consent, the cognitive (MMSE) screening will be performed with a research assistant. Individuals confirmed with no cognitive impairment will be considered enrolled. After the first visit, participants will have 1 week without any assessment or intervention training to collect accelerometer data at home under their usual conditions. They are also instructed to collect their biological samples during days when they do not have any research visits. After this is done, participants will return to continue the remaining assessments (see Table 1).

### 2.6 Sample size

A power and sample size analysis was performed by a biostatistician team independent of the study team, based on a previous meta-analysis comparing exercise training alone to combined physical and cognitive intervention on cognition (Zhu et al., 2016). Using an intent-to-treat principle with multiple imputations, a sample size of 42 subjects in each group will have 80% power to detect a group difference in means of −0.186 (the difference between a physical alone training mean of 0.239 and a combined training mean of 0.425, for an effect size of 0.624) assuming that the common standard deviation is 0.298 using a two group *t*-test with a 0.050 two-sided significance level. Taking into account a 20% attrition rate observed in our previous exercise studies, we will thus recruit 53 participants per intervention arm (53 × 3 arms), for a total of 159 participants. For the secondary outcome (neuroimaging), we based our calculation on the 2% increase in hippocampal volume following a 12-month physical exercise training reported by Erickson et al. (2011) (Erickson et al., 2011). Results suggest that 60 participants are needed to observe significant changes post-exercise. Since 2 arms involve exercise training in this study (total 106 participants), the numbers of participants are largely enough.

### 2.7 Recruitment

Participants are recruited by advertising the study to new members of the EPIC Center, reaching out to community-dwelling adults through online and printed communications (e.g., posters, videos, interviews, newsletters), phone calls to individuals who have agreed to be contacted for a research project, and by the Montreal Heart Institute’s physicians. Interested individuals’ contact information is registered in a password-protected encrypted file with restricted access.

### 2.8 Allocation

#### 2.8.1 Sequence generation

The randomization is stratified by sex (i.e., male/female) to ensure an appropriate balance of the participants’ characteristics between each training arm. The MHICC generated the randomization sequence using computer-generated random numbers sequence using permuted blocks of different undisclosed sizes.

#### 2.8.2 Concealment mechanisms

The randomization is performed by a staff member not involved in any other aspect of the trial. Once a participant is ready to be randomized the randomization staff opens a sealed envelope and reveals the training group. The participant’s intervention group is then shared with a different staff member in charge of the study training. Nobody else involved in the study has access to the randomization envelopes and they do not know any information related to the randomization procedure and sequence.

#### 2.8.3 Implementation

Individuals who provide their consent, fulfill the inclusion criteria, and complete the baseline assessment are randomized. The recruitment coordinator shares the new participant’s initials, sex, and birth date to the randomization staff, who reveals the newly assigned training group to the training coordinator.

### 2.9 Blinding

ACTIONcardioRisk is a double-blinded trial. Research personnel performing the outcome assessments at baseline, 6, and 12 months are blinded to group allocation. Participants are also blinded to the “active” intervention and study hypotheses. To maximize the blinding, all participants are informed that throughout the year everyone will have to do some tablet-based cognitive sessions (without specifying whether they are training sessions or assessments). The training groups were described in such a way that the participants could not identify the types or difference between the training groups. However, it is impossible to objectively know if at some point they found a way to guess the type of interventions available. In case of medical necessity, the participant could be unblinded, and the principal investigator will be contacted to allow the disclosure. No modification of group allocation can be made by participants or study staff during the intervention.

### 2.10 Data collection

The collection of measurements and training data throughout the study is managed by assessors and trainers using online case report forms specifically dedicated to each testing session. Medical data is collected on paper and stored separately from all other study data.

### 2.11 Data management

All physically collected data is stored in locked filing cabinets in the research center. Data from those files is entered electronically immediately after the collection by the same research assistant that receives or collects the data. All electronic data is password protected. All research data uses only the participant ID as a means of identification.

### 2.12 Statistical methods

Data analyses will be blinded to the participants’ intervention arms. All participants randomized will be included in the analyses using an intention to treat method. The variables in the study will be presented using descriptive statistics. The mean, standard deviation, median, minimum, Q1, Q3, and maximum will be analyzed as continuous variables. The number and percentage will be presented for the nominal/ordinal variables. The assumptions of the statistical tests (including normality tests) will be examined, and data transformation or non-parametric analyses may be used as appropriate, according to the guidelines (Tabachnick and Fidell, 2019). The SPSS software and SAS version 9.4 or higher software will be used to conduct the analyses.

The primary outcome will be the changes in cognitive performance (MoCA, processing speed, executive functions, and memory). Computerized cognitive tasks will be analyzed separately. We will perform four linear mixed-effects models analyses (for every cognitive measure) with intervention group as fixed factors and adjustments for relevant covariates. An unstructured covariance structure will be used to model the within-patient errors. The final selection of covariates will include age, sex, and education and any potential relevant demographic variable that show a group difference. These will consider, but not be limited to BMI, stress, mood, genetic risk, medical history, and CVRF. A Bonferroni correction will control for multiple comparisons with a significance level set at *p* < 0.0125. The same statistical model will apply for the brain imaging secondary outcomes: pulsatility, vasoreactivity and autoregulation indices, regional cerebral blood flow, grey matter volume and CSVD lesions (Bonferroni-corrected for number of regions). To inform potential future studies, exploratory analyses will also performed in which we will test if certain variable impact the effectiveness of the intervention on the primary (cognition) or secondary (imaging) variables. Due to the exploratory nature of those analyses, no multiple comparison correction will be made and no *a priori* power analysis is made. The exploratory analyses can include but not be limited to testing the mediating or moderating role of the available medical (including cardiovascular health, cardiovascular risk factors), psychological, and biological markers on the group effect. The mediating and moderating analyses will be performed according to the guidelines published by Hayes (2017).

### 2.13 Adverse events reporting

In this trial, an adverse event is defined as any medical occurrence that is unexpected and which could be reasonably suspected to be related to participation in the research study. The collection of adverse events starts after the participants’ study enrollment. It will stop with the end of their participation in the study, except in case of a serious adverse event (SAE). If the event occurs before any training session, it will be reported as non-related to the intervention. All adverse events are recorded by the study coordinator on an ongoing paper log and monitored by the physician until resolved. The physician will judge the intensity of the event and its relationship to study procedures. All adverse events that meet the SAE criteria will be reported to the local Research Ethics Board as an SAE. A SAE for this study is any major medical issue causally related to the study intervention, including hospitalization, life-threatening conditions, and severe or permanent disability.

## 3 Discussion

This project is in line with a major priority of the Canadian Institute of Aging and the WHO; to “add health to years.” This project will go beyond correlative observations by comparing the impacts of a combined intervention to physical exercise training alone on cognitive and brain function, as well as on peripheral vascular function in seniors living with CVRF. The composition of the intervention groups and the rigorous randomization procedure will help minimize bias and isolate the effect of the combined training. This project brings together experts from cognitive neuroscience, cardiology, neuropsychology, physiology, brain imaging, and kinesiology.

It is expected that the results of this multidisciplinary effort will help develop optimal and applicable approaches to prevent CVRF-induced neurocognitive deterioration. This will have a real-world impact by informing future interventions (experimental or clinical) about the ideal composition of an effective exercise or combined program targeting the average older population at risk of cognitive decline. Moreover, the neuroimaging component will be able to document the neurovascular mechanisms through which the intervention could improve cognition by talking into account each participant’s individual profile (genetic profile, physiological markers of cardiovascular health, and psychological profile). Exploratory pathways that could also modulate the impact of the training arms on cognition are also considered, which include diet patterns, sleep quality, cognitive reserve, chronic stress, and mood.
